# Could the Convergence of Science and Technology Guarantee Human Health in the Future?

**DOI:** 10.3390/bioengineering10050589

**Published:** 2023-05-13

**Authors:** Ali Zarrabi

**Affiliations:** Department of Biomedical Engineering, Faculty of Engineering & Natural Sciences, Istinye University, 34396 Istanbul, Türkiye; ali.zarrabi@istinye.edu.tr or alizarrabi@gmail.com

Due to the daily growth of the world population, there has been an increase in concerns regarding health, especially due to the increase in the number of aged people, the surge of pollution, and the appearance of new pandemic diseases such as COVID-19 and influenza H1N1. One exciting approaches that provides much hope is the convergence of science and technology, which can improve the performance of routinely used strategies (in both diagnosis and treatment) and even create new approaches for monitoring the healthcare of the global population. This could be put into practice by creating new therapeutic compounds against cancer, which could target the intracellular pathways [1], or by producing new nanomaterials that could carry therapeutic compounds [2]. The convergence of science and technology could also be used for the creation of low-cost highly sensitive biosensors used for point-of-care applications, such as monitoring heart rates [3], detecting therapeutic compounds, such as antibiotics in pharmaceutical and clinical samples [4], and as highly sensitive and selective aptasensors [5]. This convergence could also help overcome the problem of low efficiency in conventional in vitro and in vivo models in the development and assessment of new drug formulation using different types of organ-on-chip models [6]. In this context, Osouli Tabrizi and his coworkers represented a type of cells-on-a-chip platform for the modeling of complementary metal oxide semiconductors, as a low-cost sensor for the detection of living cells, which could help detect periodontal diseases early and with high accuracy. The results of this study showed the effectiveness of this new sensor in monitoring the cells in very small sample saliva volumes (1 µL) [7]. In another study, the application of hydrogels containing drug, cells, and growth factor for the treatment of osteoporosis showed promising signs of improvement in comparison to implants and metallic scaffolds [8]. On the other hand, coating the currently used stainless-steel (SS) implants with a polymeric shell of zein/Ag-Sr doped mesoporous bioactive glass nanoparticles has reduced the toxicity effects of the SS implants and exhibited beneficial effects in bone regeneration applications [9]. In another study, Sikder et al. evaluated the combination of ultrasound (US) and the 3D-printed bioactive piezoelectric scaffolds for the treatment of orthopedic defects. They have shown that the fabricated scaffold that contained bioactive PCL-BaTiO_3_ piezoelectric composite formulations could improve osteogenesis. On the other hand, the application of US in combination with this scaffold could induce pre-osteoblast adhesion, proliferation, spreading (at 1 Hz), and osteoblast differentiation (at 3 HZ) [10]. In a recent study, Vieira et al. introduced a new type of theranostic platform based on gellan gum hydrogel functionalized with Mn molecules, which showed T1-weighted MRI features, and at the same time, the capability of delivering stem cells to the central nervous system (CNS) in a noninvasive manner. Therefore, the combination of hydrogel-based formulation, Mn, and MRI technology resulted in the real-time monitoring of cell delivery to the CNS system [11]. Another interesting direction that the simultaneous application of science and engineering can take is the utilization of microorganisms for removing pollutant from the environment, while simultaneously producing useful materials. We have shown that *Chlorella vulgaris* (*C. vulgaris*), as a type of microalgea, has the capability of removing antibiotic contamination from the water via a 3-steps bioreaction, bio-adsorption, bioaccumulation, and biodegradation. In addition, this study shows that the microalgae produce some useful byproducts, such as proteins, lipids, chlorophyll-a, and carbohydrates [12].

Recently, with the emergence of artificial intelligence (AI) and the rapid growth of its application in different fields of medicine, there is renewed hope for the fast detection of diseases and the prediction of the effectiveness of the performance of new drugs and therapeutic methods, which will help professionals make better and more informed decisions [13,14]. Computational modeling is one of the subcategories of AI that could be used, in combination with in vitro tests, to predict and evaluate the results of different treatments. This could reduce the time of treatment and improve its performance. We have used the in silico 3D and single cell ventricle simulations to evaluate the effects of mexiletine on cardiac cells, and the results showed that this drug could reduce the action potential duration (APD) in a long QT variant 3 (LQT3) patients with an A1656D mutation, shift the occurrence of alternants from a normal heart rate in the cells to a quicker one, and eliminate the possibility of a breakup of the spiral wave [15]. In another study, *Arippa* et al. used computational modeling for the evaluation of the kinematic parameters related to Parkinson’s disease (PD). They selected “61 people with PD (aged 68.9 ± 9.3 years) and 47 unaffected individuals age- and sex-matched (66.0 ± 8.3 years)” to evaluate the differences between them in angular trends at hip, knee, and ankle joints by applying the “computerized 3D gait analysis performed using an optical motion-capture system”. They presented a new approach for the early diagnosis of PD since, according to their results, the patients had significant alterations in interlimb coordination, which could be detected at the hip and ankle joints and had “a modified gait pattern particularly at the terminal stance/early swing phase of the gait cycle” [16].

According to the aforementioned research, this Special Issue aims to highlight the beneficial effects of the convergence of science and technology in different aspects of human health, from the daily monitoring of health to the introduction of novel or improved treatment and diagnosis methods for different types of disorders, which could be helpful in tackling the current challenges health management among nations.

## Data Availability

No new data were created or analyzed in this study. Data sharing is not applicable to this article.
